# Rudimentary third lower limb in association with spinal dysraphism: Two cases

**DOI:** 10.4103/0019-5413.30530

**Published:** 2007

**Authors:** Ashish P Wasnik, Atul Shinagare, Usha R Lalchandani, Rahul Gujrathi, Bhujang U Pai

**Affiliations:** Department of Radiology, Grant Medical College and Sir J.J Group of Hospital, Byculla, Mumbai - 400 008, India

**Keywords:** Rudimentary/accessory third limb, spinal dysraphism, tripagus

## Abstract

Spinal dysraphism is a common congenital anomaly with many associated variants. One of the rarest associated findings is a full grown or rudimentary third limb, collectively called Tripagus. We present two cases of spinal dysraphism with rudimentary third limb arising from the ilium.

Defects in the early embryonic stages produce spinal dysraphism, which is characterized by anomalous differentiation and fusion of dorsal midline structures. Spinal dysraphism may be categorized clinically into two subsets, open spinal dysraphism and closed spinal dysraphism. Rudimentary accessory (third) limb is a very rare anomaly seen in association with spinal dysraphism. We present two such cases.

## CASE REPORTS

### Case 1

A 11-year-old boy presented with progressive weakness in the right lower limb and a soft swelling over the lower back since birth. Patient had history of urinary incontinence. Examination revealed a soft, nontender, compressible immobile swelling and presence of two nondischarging dermal sinuses adjacent to the swelling.

Radiographs of the lumbosacral spine showed presence of a spina bifida at the L4 and L5 segments and a posterior soft tissue mass.

Computed tomography (CT) confirmed findings of spina bifida along with presence of a lipomyelomeningocele and two dermal sinuses. A bony strut was noted arising from the left ilium, extending upward. It had two long bone segments, the longer segment measured approximately 6.5cm and the shorter segment approx 1.5cm, with a rudimentary joint between them. No other anorectal or urogenital abnormalities were detected.

**Figure 1:**
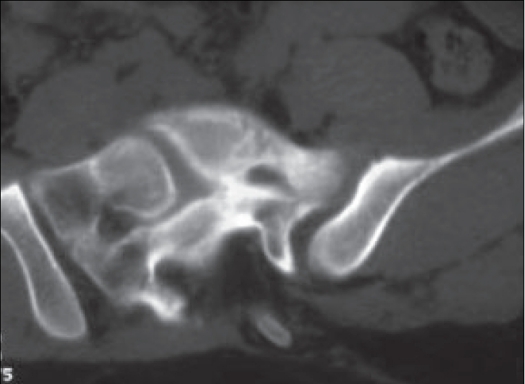
MRI showing the bony strut with spinal dysraphism, lipomyelomeningocele and dermal sinus

Magnetic resonance imaging (MRI) confirmed these findings of lipomeningocele with dermal sinus and bony strut. These bony structures may represent a developmentally abortive accessory limb [Figure 1].

### Case 2

A nine-year-old boy presented with complaints of progressive difficulty in walking, urinary incontinence and deformity and nonhealing ulcers of the feet since past one year and a history of a swelling over the sacral region present since birth, which was constant in size.

On examination, he had weakness in both his lower limbs, muscle wasting of both legs (more on the left side) and his left foot had an equinus deformity. The swelling over his sacral region was soft, compressible and nonmobile. No other cutaneous stigmata were present. He had trophic ulcers over his buttocks and over pressure points of his feet.

**Figure 2:**
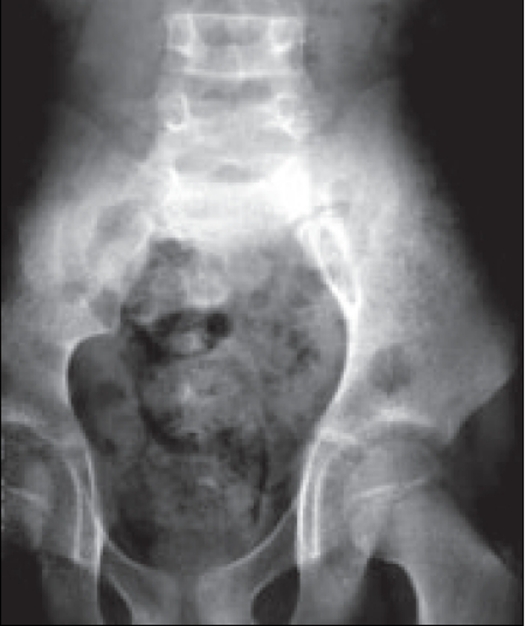
Frontal radiograph showing absence of left sacrum(Scimitar sacrum)-Case 2

Plain radiograph of the lumbosacral spine [Figure 2] showed absence of the left side of the sacrum (Scimitar sacrum) and spina bifida of the L5 vertebra. Soft tissue swelling over the sacrum was also visible.

**Figure 3:**
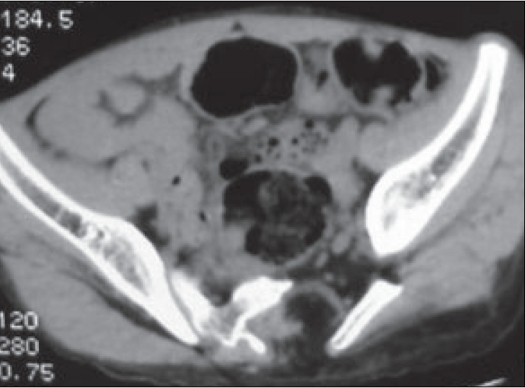
Axial CT showing absent left hemi sacrum with bony strutarising from the ilium

**Figure 4:**
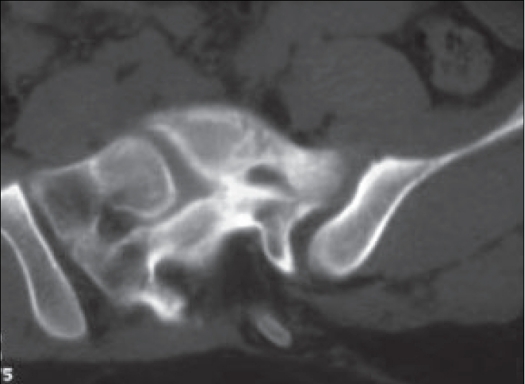
Axial CT scan showing spinal dyraphism and the hypodensesinus tract

CT confirmed the L5 spina bifida and absence of the left half of the sacrum. A hypodense soft tissue of fat attenuation was seen extending from the spinal canal into the soft tissue swelling over the sacrum (lipomeningocele). A bony strut was seen to arise from the left iliac blade measuring approximately 5cm, which could represent a rudimentary limb [Figures 3 and 4]. No anorectal or urogenital abnormality was evident.

**Figure 5:**
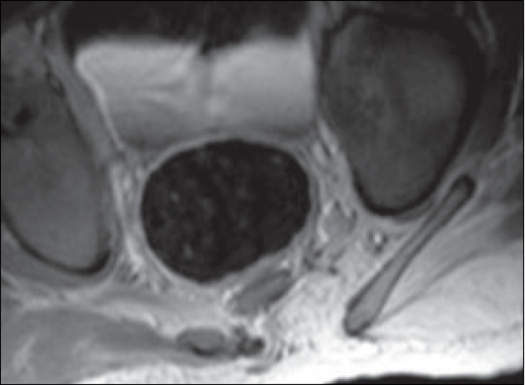
Axial T1W MRI showing the bony strut with spinal dyraphism

**Figure 6:**
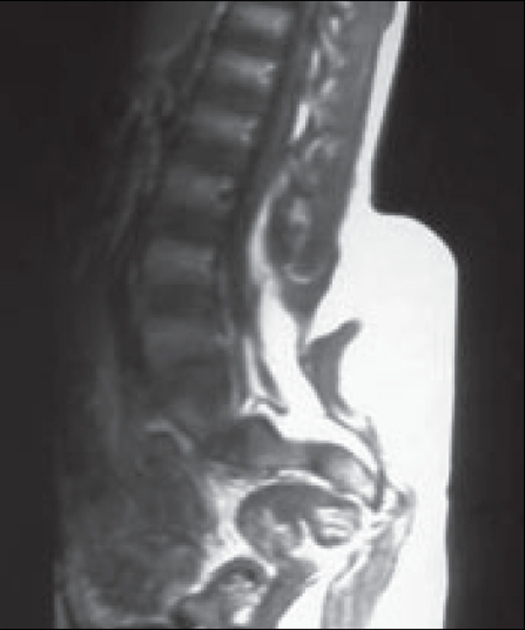
Saggittal T1W MRI showing large lipoma extending subcutaneously through the bony defect. Segment of the rudimentary accessory limb also seen

The MRI scan performed with T1WI and T2WI sequence in the saggittal, coronal and axial plane revealed spinal dysraphism with a large intraspinal lipoma merging with the dorsal aspect of the cord and continuous with the subcutaneous lipoma. The spinal cord appeared to be terminating in this lipomatous mass. This lipoma was also extending subcutaneously through the spina bifida defect. A bony structure with cortex and medulla was seen to arise from the left iliac blade extending downward, which could represent a developmentally aborted third limb [Figures 5 and 6].

## DISCUSSION

Spinal dysraphism is a generic term describing pathologic conditions related to improper closure of the caudal neuropore. It encompasses all conditions associated with spina bifida. Spinal dysraphisms result from derangement in the normal embryogenetic cascade occurring during a limited period of time during early embryonic development, between the second and sixth gestational weeks. Gastrulation (weeks 2-3), primary neurulation (weeks 3-4) and secondary neurulation and retrogressive differentiation (weeks 5-6) are the steps involved.

Spinal dysraphism may be divided into two groups. Open spinal dysraphism are always associated with a Chiari II malformation and include myelomeningocele, myeloschisis, hemimyelomeningocele and hemimyelocele. Closed spinal dysraphism are covered by intact skin, although cutaneous stigmata may be present. Two subsets may be identified based on whether a subcutaneous mass is present in the low back. Closed spinal dysraphisms with mass comprise lipomyeloschisis, lipomyelomeningocele, meningocele and myelocystocele. Closed spinal dysraphism without mass comprise complex dysraphic states (ranging from complete dorsal enteric fistula to neurenteric cysts, split cord malformations, dermal sinuses, caudal regression and spinal segmental dysgenesis), bony spina bifida, tight filum terminale, filar and intradural lipomas and persistent terminal ventricle.

A full grown or rudimentary third limb is a very rare association seen with spinal dysraphism and few cases are mentioned in the literature^1^–^5^ but its exact incidence is unknown. Various associations of urogenital duplication, anorectal malformation and other anomalies have been found.^6^

Accessory limb may be a result of very early splitting of the limb bud arising from the paraxial mesoderm. It is proposed that the growth of the accessory limb occurs from a mesodermal blastema that is a result of de-differentiation from Schwann cells.^2^ A primary mesodermal defect involving both the limb bud and the adjacent para-axial mesoderm may explain the association of this anomaly with spina bifida.^7^ A possibility of twinning has also been postulated in an accessory limb arm.^8^

Developmentally aborted accessory limb may be an incidental finding in a patient presenting with neurological or other complaints associated with spinal dysraphism.

In cases like ours, diagnosis of an abortive limb is mainly radiological as there may be no clinical sign to suggest its presence. It may be seen on plain radiograph as a bony strut arising from pelvic bones. It is well appreciated on CT or MRI. These bones have well formed cortex and medulla and follow the intensity of normal bones on MRI. Rudimentary joint may be present as well. There may be muscles attached to these bones.

MRI is the modality of choice, as it can assess other associated conditions like meningocele, myelomeningocele, lipomeningocele, split cord malformations, dermal sinuses and associated anomalies. Treatment in the form of surgical correction has been mentioned in cases with completely developed third limb. It includes excision of the accessory limb or at times disarticulation and reconstruction of the accessory limb which has no innervation with removal of one set of duplicated lower genitourinary and alimentary tracts with reconstruction of the pelvis.^9^ In a case of dipegus, mentioned in the literature, with three accessory legs, the parasitic pelvis and rest of the legs were extirpated with an attempt to correct the associated anorectal or genitourinary malformation.^6^

The exact role of surgery in rudimentary or aborted accessory limb as in our case, where there was no cosmetic defect or neurological manifestation due to it, remains questionable.
